# Laboratory Analysis of Tularemia in Wild-Trapped, Commercially Traded Prairie Dogs, Texas, 2002

**DOI:** 10.3201/eid1003.030504

**Published:** 2004-03

**Authors:** Jeannine M. Petersen, Martin E. Schriefer, Leon G. Carter, Yan Zhou, Tara Sealy, Darcy Bawiec, Brook Yockey, Sandra Urich, Nordin S. Zeidner, Swati Avashia, Jacob L. Kool, Jan Buck, Connie Lindley, Leos Celeda, John A. Monteneiri, Kenneth L. Gage, May C. Chu

**Affiliations:** * Centers for Disease Control and Prevention, Fort Collins, Colorado, USA; † Centers for Disease Control and Prevention, Atlanta, Georgia, USA; ‡ Texas Dept of Health, Arlington, Texas, USA; § State Veterinary Administration, Prague, Czech Republic

**Keywords:** *Francisella tularensis*, tularemia, oropharyngeal, *Cynomys ludovicianus*, prairie dogs, outbreak, exotic pets, seropositive, culture-positive

## Abstract

Oropharyngeal tularemia was identified as the cause of a die-off in captured wild prairie dogs at a commercial exotic animal facility in Texas. From this point source, *Francisella tularensis*–infected prairie dogs were traced to animals distributed to the Czech Republic and to a Texas pet shop. *F. tularensis* culture isolates were recovered tissue specimens from 63 prairie dogs, including one each from the secondary distribution sites. Molecular and biochemical subtyping indicated that all isolates were *F. tularensis* subsp. *holarctica* (Type B). Microagglutination assays detected antibodies against *F. tularensis,* with titers as great as 1:4,096 in some live animals. All seropositive animals remained culture positive, suggesting that prairie dogs may act as chronic carriers of *F. tularensis*. These findings demonstrate the need for additional studies of tularemia in prairie dogs, given the seriousness of the resulting disease, the fact that prairie dogs are sold commercially as pets, and the risk for pet-to-human transmission.

*Francisella tularensis* is the causative agent of the zoonotic disease tularemia (*1*,*2*). As few as 10 organisms are sufficient to cause severe disease and death, making *F. tularensis* one of the most infectious bacterial pathogens known. Thus, *F. tularensis* is considered to be a biological threat agent that poses a substantial risk to public health (*3*).

Infections with *F. tularensis* are widely distributed and occur in >100 wildlife species in the Northern Hemisphere (*4*,*5*). Two subspecies of *F. tularensis* are most commonly associated with human and animal disease: *tularensis* (Type A) and *holarctica* (Type B) (*6*,*7*). Type A is found almost exclusively in North America and is associated with a severe form of disease in humans and rabbits (*Lepus* spp.). It is commonly differentiated from Type B by its ability to produce acid from glycerol. Type B is found throughout the Northern Hemisphere (holarctic region); it does not produce acid from glycerol and rarely causes death in humans. Type B is most frequently isolated from rodent species, including muskrats (*Ondatra zibethicus*), mice (*Mus musculus*), beaver (*Castor canadensis*), voles (*Microtus* spp.), and water voles (*Arvicola terrestris*).

Infections with *F. tularensis* also occur in the black-footed prairie dog (*Cynomys ludovicianus*) (*8*). This finding has particular public health significance since wild-caught prairie dogs are sold as pets both domestically and internationally. Wild prairie dogs are found throughout the Great Plains of North America from southern Canada to just inside Mexico. Every year, pups are collected in the United States during April through July and are distributed to pet stores throughout the country as well as being exported internationally.

The first literature report of tularemia in captive prairie dogs described *F. tularensis* infection in three wild-caught animals in 1986 (*8*). Subsequently, *F. tularensis* infection caused by Type B was confirmed by the Centers for Disease Control (CDC), Fort Collins, Colorado, in wild-caught prairie dogs, originating from on animal exporter and shipped to research institutions in Boston and Houston from 1996 to 1997. In the summer of 2000, CDC again confirmed Type B infection in a wild-caught prairie dog. In this case, a family traveling from Ohio purchased two prairie dogs from a dealer in Kansas; one animal died during transport, while the second animal displayed disease and died after they arrived home.

In August of 2002, an outbreak of tularemia was identified as the cause of a die-off among wild-caught, commercially traded prairie dogs at an exotic animal facility in Texas. We describe laboratory findings from this investigation. The epidemiologic findings of the investigation are reported separately (*9*). During this outbreak, many animals died of infection with *F. tularensis*. However, a small number of surviving animals developed antibodies against *F. tularensis*, suggesting that prairie dogs can survive an infection of tularemia. All seropositive animals were also found to harbor live infectious bacteria, suggesting that prairie dogs may be persistently infected. These findings have important public health implications in light of commercial prairie dog trade practices.

## Materials and Methods

### Outbreak Groupings

On August 2, 2002, a total of 163 prairie dogs were found at the exotic animal facility in Texas. These animals were classified into four groups: group A (bin 1, dead), group B (bin 1, live), group C (escapees), and group D (bin 2 and cages, healthy). Group A animals (n = 46) were collected during the last week of July through August 2, 2002. All group A animals had been housed in an uncovered metal tub (bin 1). The live animals remaining in bin 1 were classified as group B (n = 23), with most of the animals being emaciated, dehydrated, and lethargic. Group C (n = 36) comprised escaped prairie dogs that were running free throughout the facility. Group D prairie dogs (n = 58) were physically separated from both group B and C animals, and all group D animals were large, well-nourished, energetic, and noisy. Group D animals were housed in an uncovered metal tub (bin 2) and in several wire cages.

Animals from the Texas facility that had been sent to other locations made up two additional groups. Group E animals comprised seven prairie dogs that originated from the Texas facility, were distributed to pet shops in Texas, and recalled once *F. tularensis* was identified as the cause of the outbreak. Group F comprised 100 prairie dogs shipped from the Texas facility to the Czech Republic.

### Culture Recovery of *F. tularensis*

All prairie dogs at the Texas facility (n = 163) were necropsied on site, and tissues were surgically removed. Appropriate biosafety measures were adhered to, including the use of closed front gowns, N95 masks, glasses, and gloves. Spleen and liver samples were spread onto cysteine heart agar supplemented with 9% sheep blood (CHAB). Plates were sealed with parafilm and transported in ice coolers (~15°C–20°C) until arrival at the CDC laboratory, Fort Collins, Colorado (~72 hours). Culture plates were then transferred to a biosafety level (BSL) 3 incubator at 37°C for 5 days and checked daily for *F. tularensis* growth. Some tissues were also spread onto CHAB medium containing antibiotics (*10*), incubated at 37°C for 7 days, and checked daily for *F. tularensis* growth. A culture isolate from prairie dogs shipped to the Czech Republic was grown at the State Veterinary Administration, Prague, Czech Republic, and submitted to our laboratory.

Spleen and liver tissues were injected into pathogen-free Swiss-Webster outbred mice for culture recovery of *F. tularensis* (IACUC Protocol 00-06-018-MUS). Tissues (~1 gm) from individual prairie dogs were ground with mortar and pestle, resuspended in 2 mL of saline and 0.5 mL of the tissue suspension was injected subcutaneously (s.c.) into mouse. All injections were performed in a BSL2 animal facility, and appropriate biosafety measures were followed, including the use of closed front gowns, N95 masks, glasses, and gloves. Animals were euthanized when signs and symptoms of tularemia were evident. After euthanasia was performed, 0.5 to 1.0 mL of whole blood was removed by cardiac puncture with a 1.0 mL tuberculin syringe. Liver and spleen tissues were surgically removed and spread onto CHAB with sterile wooden sticks. All healthy injected mice were euthanized 21 days after injection, and serum was tested for anti-*F. tularensis* antibody.

### Direct Fluorescent Assay (DFA)

Slide touch preparations of tissues were prepared and heat-fixed immediately after necropsy at the Texas animal facility. On arrival at the laboratory, all slides were incubated with FITC-labeled rabbit anti-*F. tularensis* subsp. *tularensis* (SchuS4 strain) antibodies (CDC) for 30 minutes at room temperature. Slides were washed twice in phosphate-buffered saline, followed by a final rinse with dH_2_O and viewed with a fluorescent microscope using the 40X objective and a 490 nm filter. Direct fluorescent activity was scored independently by two technicians experienced with *F. tularensis* DFA.

### Serologic Findings

For all group B, C, and D animals, blood samples were collected from euthanized animals by cardiac puncture. Blood was collected into Microtainer brand serum separator tubes (Becton Dickinson, Franklin Lakes, NJ) and maintained at 4°C until arrival at the laboratory (~72 h). Serum was separated, heat-inactivated for 30 min at 56°C, and tested for *F. tularensis* specific antibodies by using a standard microagglutination assay (*11*). Briefly, serial dilutions of serum were incubated overnight with safranin-stained, formalin-killed *F. tularensis* subsp. *tularensis* (SchuS4 strain) cells at room temperature, and a titer was assigned reflecting the last well demonstrated full agglutination. Samples with a titer of 1:128 or greater were reported as positive.

### Confirming *F. tularensis*

Prairie dogs were confirmed positive on recovery of an isolate with characteristic growth on CHAB and positive results by DFA or IS*Ftu2* polymerase chain reaction (PCR). Animals were considered presumptive positive if tissues tested positive by DFA or PCR, but no isolate was obtained. Prairie dogs were considered negative if all three diagnostic tests (culture, DFA, serologic testing) failed to detect any evidence of *F. tularensis* infection. For negative samples, recovery of culture included passage of the spleen and liver tissues through mice injected with *F. tularensis*.

### *F. tularensis* Subtyping

For molecular subtyping, DNA was prepared after injection of a 1 μL loop of culture into 200 μL TE buffer. Cells were lysed by boiling at 95°C for 10 min. A differential PCR, based on the presence or absence of the IS*Ftu2* element (GenBank accession no. AY06), was performed by using 1 μL of the lysed bacterial supernatant and the primers TuF1705 (5′-GATAGATACACGCCTTGCTCACA-3′) and TuBR431(5′-ACCCAGCCAATGCCTAAATA-3′) (Y. Zhou, unpub. data). The amplification program included a denaturation cycle at 95°C for 2 min, followed by 35 amplification cycles of 95°C for 30 s, 55°C for 30 s, and 72°C for 1 min, and a final elongation cycle of 72°C for 5 min. PCR products were analyzed by agarose gel electrophoresis, followed by staining with EtBr and visualization with a Bio-Rad Gel Doc UV system (Bio-Rad Laboratories, Hercules, CA). For biochemical subtyping, the 96-well automated MicroLog MicroStation System with GN2 Microplates (Biolog Inc, Hayward, CA) was used. Microplates were set up and analyzed per the manufacturer’s instructions.

### Statistical Analysis

McNemar’s test was used for statistical analysis. Sensitivities of different diagnostic tests were evaluated for their ability to detect *F. tularensis* in a given population of animals (either live or dead animals).

## Results

### Laboratory Findings

*F. tularensis*–infected prairie dogs from the Texas animal facility were traced to Texas pet shops and animals shipped to the Czech Republic. From these three sources, 177 prairie dogs (1 animal whose illness initiated the investigation [12], 163 animals that remained onsite at the Texas facility, 7 animals recalled from Texas pet shops, and 6 animals shipped to the Czech Republic) were tested. Of these animals, 63 were confirmed positive, 13 were identified as presumptive positives, and 101 were confirmed negative for *F. tularensis* infection (Table 1).

**Table 1:** Laboratory results for outbreak of tularemia in wild-trapped, commercially sold prairie dogs

Group	Prairie dogs	No. of animals	Presumptive-positive samples^a^	Confirmed-positive samples^b^	Confirmed-negative samples^c^
A	Exotic animal facility, Texas, bin 1, dead animals	47^d^	7	40	0
B	Exotic animal facility, Texas, bin 1, live animals	23	0	20	3
C	Exotic animal facility, Texas, escapees	36	0	1	35
D	Exotic animal facility, Texas bin 2 and cages, healthy	58	0	0	58
E	Pet shop recalls, originating from exotic animal facility, Texas	7	1	1	5
F	Czech Republic, originating from exotic animal facility, Texas	100	5	1	Not determined

^a^Prairie dogs were confirmed positive on recovery of an isolate with characteristic growth on cysteine heart agar with 9% sheep blood and positive testing of the isolate by direct fluorescent assay (DFA) or IS*Ftu2* polymerase chain reaction (PCR). ^b^Prairie dogs were considered presumptive positive if primary tissues tested positive by DFA or PCR but no isolate was obtained. ^c^Prairie dogs were confirmed negative if all three diagnostic tests (culture, DFA, serologic testing) failed to detect any evidence of *Francisella tularensis* infection. ^d^46 animals that remained on site August 2, 2002, plus 1 animal that initiated the outbreak investigation (TX021935)

### *F. tularensis* Isolates from Infected Prairie Dogs

Because prairie dogs were sold commercially as pets and the risk for pet-to-human transmission was unknown, determining which groups (A–D) of animals were potentially infectious was important. Subtyping the *F. tularensis* isolates was also important, since this outbreak carried the threat of international dissemination. Therefore, our laboratory efforts focused on recovery of viable organisms. In total, 63 isolates were recovered (Table 1): 61 from prairie dogs at the Texas facility (groups A–C), 1 isolate from prairie dogs recalled from Texas pet shops (group E), and 1 isolate from prairie dogs distributed to the Czech Republic (group F).

### Biochemical and Molecular Typing of *F. tularensis* Isolates

For subtyping of the 63 *F. tularensis* isolates (63 isolates described here, including 1 isolate that initiated the investigation, TX021935 [*12*]), a combination of biochemical and molecular typing was used. Biochemical characterization was performed on 15 isolates representative of all five groups of *F. tularensis–*positive animals (groups A, B, C, E, F). All 15 *F. tularensis* isolates were unable to use glycerol as a carbon source and thus were classified as Type B (data not shown). In addition, six representative isolates were tested for antimicrobial susceptibilities and demonstrated MICs consistent with those published previously for Type B (data not shown, *13*).

**Figure 1:**
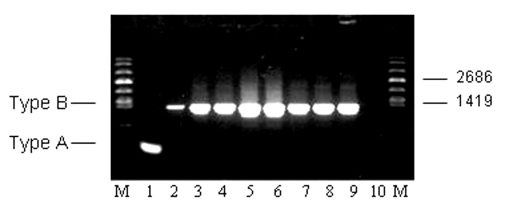
Molecular subtyping of representative *Francisella tularensis* isolates from Groups A, B, C, E, and F prairie dogs. The expected size PCR fragments for *F. tularensis* subsp. *tularensis* (Type A) and *holarctica* (Type B) are shown in lanes 1 and 2, respectively. Subtyping results for the five groups (A, B, C, E, F) are shown in lanes 3–9. Lane 3: TX021935 (A); lane 4: TX022151 (A); lane 5: TX022537 (B); lane 6: TX022592 (B); lane 7: TX022799 (C); lane 8: TX022107 (E); lane 9: CZ024233 (F). Lane 10: no DNA template control. Lane M: molecular weight markers.

To distinguish molecularly between Type A and Type B, a differential PCR based on the presence or the absence of the IS*Ftu2* element was performed (Y. Zhou, unpub. data). For Type A, a PCR product of 390 bp was amplified, whereas for Type B, a product of 1,249 bp was amplified. When IS*Ftu2* PCR subtyping was performed on all 63 isolates, all were shown to be Type B, including the single isolate received from the Czech Republic. Representative IS*Ftu2* PCR subtyping for the five groups (A, B, C, E, and F) of *F. tularensis*–positive animals is shown in Figure 1. Additional analysis with IS*Ftu2* restriction fragment length polymorphisms southern blotting demonstrated that the *F. tularensis* isolates were molecularly indistinguishable (data not shown).

### Texas Facility Investigation (Groups A–D)

The animals remaining at the Texas facility (groups A–D) provided insight into how tularemia was transmitted among the prairie dogs. When necropsies were performed on animals in groups A–D, cannibalization, as indicated by partially eaten prairie dog carcasses, was noted among group A animals. In addition, all group A and most of group B animals displayed swollen submandibular lymph nodes, suggesting that all animals ingested the bacteria.

Because all prairie dogs from the Texas facility (groups A–D) were tested and classified as confirmed positive, presumptive positive, or negative (Table 1), diagnostic test sensitivities could be determined. From the 68 prairie dogs at the Texas facility that tested positive for *F. tularensis* by one or more diagnostic methods (culture, DFA, serologic testing), 61 isolates were recovered, yielding an overall culture recovery rate of 89.7%.

### Detecting *F. tularensis* in Live, Infected Animals

For determining *F. tularensis* infection in live, infected animals, the sensitivities of culture versus DFA and serologic testing were compared (Table 2). Testing all 59 animals in groups B and C, confirmed 21 animals as *F. tularensis–*positive and 38 animals as *F. tularensis–*negative (Table 1). For the 20 animals confirmed positive by analysis of spleen and liver tissues, culture detected *F. tularensis* in 100% of cases. In contrast, both DFA and serologic testing detected *F. tularensis* in 10 of 20 animals, yielding a sensitivity of only 50% (Table 2). These differences were significant (p < 0.05) and demonstrate that culture of spleen and liver tissues is more sensitive than DFA or serologic testing for detecting *F. tularensis* in live, infected prairie dogs.

**Table 2:** Comparison of diagnostic sensitivities of culture and direct fluorescent assay (DFA) for detection of *Francisella tularensis* in live versus dead prairie dogs (groups A–C)

Prairie dogs^a^	No. (%) of samples positive for:			
	Culture (spleen/liver)	Direct fluorescence (spleen/liver)	Direct fluorescence (lymph node)	Serologic testing
Groups B, C; live, infected animals (n = 20)	20 (100)	10 (50)	17 (89.5)^b^	10 (50)
Group A, dead animals (n = 47)	40 (85.1)	47 (100)	Not tested	Not tested

^a^All 67 prairie dogs tested positive for *F. tularensis* by at least one diagnostic test (culture, DFA, or serologic testing). ^b^19 *F. tularensis*–positive animals were tested.

Since the outbreak was consistent with oropharyngeal tularemia, submandibular lymph nodes of group B animals were also analyzed. When lymph nodes were cultured, an additional case was confirmed by isolation of *F. tularensis* from animal B17 (Table 3). The bacterium was not cultured from the spleen and liver of this prairie dog even on passage of tissues through Swiss-Webster mice. This finding suggested that prairie dog B17 had recently ingested *F. tularensis* and that the infection was localized to the submandibular lymph nodes.

**Table 3:** Diagnostic test results for culture–positive group B prairie dogs^a^

Prairie dog	DFA (spleen/liver)	DFA (submandibular lymph node)	Serologic testing (microagglutination assay)	
B1	+	+	1:512	Pos
B2	+	+	1:32	Neg
B3	+	+	1:8	Neg
B4	–	+	1:1,024	Pos
B5	–	+	1:4,096	Pos
B6	+	+	1:512	Pos
B7	–	+	1:512	Pos
B8	+	No sample	1:8	Neg
B10	+	+	1:4	Neg
B11	–	+	1:256	Pos
B12	–	–	1:1,024	Pos
B13	–	–	0	Neg
B14	–	+	1:128	Pos
B15	+	+	1:64	Neg
B16	+	+	1:16	Neg
B17	–	+	0	Neg
B18	+	+	1:512	Pos
B19	+	+	1:4	Neg
B20	–	+	1:128	Pos
B21	–	+	1:16	Neg

^a^DFA, direct fluorescent assay; Neg, negative; Pos, positive.

To determine if lymph node tissues were a better tissue source than either spleen or liver tissues for detection of *F. tularensis*, DFA was used for direct comparison of tissues from culture-positive group B animals (Table 2). When spleen and liver tissues were analyzed, the sensitivity of DFA was 50%, whereas for analysis of submandibular lymph node tissues the sensitivity of DFA was 89.5% (Table 2). This difference in sensitivities was significant (p < 0.05) and demonstrates that for cases of oropharyngeal tularemia, submandibular lymph node tissues are the most appropriate source for detecting infection by DFA.

### Detecting *F. tularensis* in Fatal Cases of Tularemia

For fatal cases of tularemia, the sensitivity of culture and DFA was also compared (Table 2). Of the 47 animals in group A, 40 were confirmed positive, and 7 were presumptive positive for *F. tularensis* (Table 1). Direct fluorescence analysis of spleen and liver tissues identified all 47 animals as *F. tularensis* positive, yielding a sensitivity of 100%. In contrast, 40 *F. tularensis* isolates were obtained, yielding a sensitivity of 85.1%. These results were significant and demonstrated that DFA was more sensitive than culture for detection of *F. tularensis* in carcasses (p < 0.05).

### Seropositivity and Decreased *F. tularensis* Levels in Live, Infected Animals

To test for evidence of seroconversion in live, infected animals, serum samples from group B prairie dogs were checked for anti-*F. tularensis* antibodies. Ten animals showed evidence of seroconversion, displaying titers against *F. tularensis* as great as 1:4,096 (Table 3). To our knowledge, this evidence is the first that prairie dogs can develop specific antibodies on infection with *F. tularensis*. In addition, *F. tularensis* was successfully recovered from the spleen of all 10 seropositive animals, suggesting that prairie dogs may become persistently infected.

**Figure 2:**
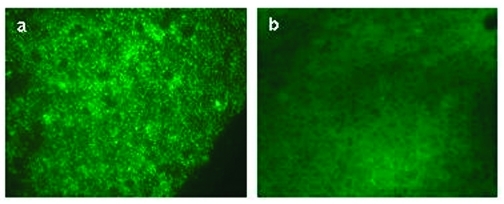
Direct fluorescent assay (DFA) results on spleen tissues from a seronegative (panel a) and seropositive (panel b) prairie dog.

Comparison of DFA results for seropositive and seronegative prairie dogs indicated that the levels of *F. tularensis* in liver and spleen were greatly decreased in seropositive prairie dogs. In 7 of 10 seropositive prairie dogs, *F. tularensis* was not detectable by DFA analysis of spleen and liver tissues (Table 3, Figure 2, panel b). Conversely, 7 of 10 seronegative animals were positive by DFA analysis of spleen and liver tissues (Table 3, Figure 2, panel a). These findings demonstrate that seropositivity in prairie dogs leads to decreased levels of *F. tularensis* and may suggest that seropositive prairie dogs can survive an acute infection of oropharyngeal tularemia.

## Discussion

In our study, we documented the laboratory results from an outbreak of oropharyngeal tularemia among wild-caught, commercially distributed prairie dogs. *F. tularensis*–infected prairie dogs from the Texas animal facility were traced to Texas pet shops and to the Czech Republic. Our findings indicate that the primary mechanism of transmission was ingestion of *F. tularensis*, as all infected prairie dogs displayed enlarged submandibular lymph nodes, a hallmark of oropharyngeal tularemia. In addition, all prairie dogs (group D) physically separated from sick animals were negative for *F. tularensis* infection, demonstrating that the outbreak of tularemia at the Texas facility required contact with infected animals. While other modes of bacterial ingestion cannot be ruled out, this outbreak most likely resulted from cannibalism of dead animals. Cannibalism, as evidenced by partially eaten carcasses, was observed at the Texas facility as well as in the shipment of animals to the Czech Republic. In nature, cannibalism occurs in rodents and has been previously documented as the cause for spread of tularemia (*14*,*15*). Studies of the black-tailed prairie dog in nature have also documented cannibalism (*16*). At the Texas exotic animal facility, group A and B animals were placed together in a single metal bin, which allowed unnaturally close contact and conditions. Also, the use of wood chip bedding increased the likelihood that buried carcasses would not be seen, probably contributing to delayed removal of deceased animals, thereby increasing the opportunity for cannibalism.

Since, *F. tularensis* in pet prairie dogs presented an unaddressed public health threat for their owners, we focused our efforts on the recovery of live organisms. We were able to culture infectious bacteria from both dead and live, infected animals. Moreover, our study is the first to provide evidence that prairie dogs can develop antibodies against *F. tularensis*. The seropositive prairie dogs might have survived long-term, since these animals had decreased levels of infecting bacteria and were blood-culture negative (unpub. data). These findings raise the possibility that persistent infection occurs in prairie dogs and suggests a potential role of prairie dogs as reservoirs of *F. tularensis* in nature. Our findings with prairie dogs are very similar to previous reports documenting chronic infection in seropositive voles infected orally with *F. tularensis* (*17*,*18*). In one of those studies, seropositive voles were shown to harbor live *F. tularensis* for as long as 313 days.

Although culture is considered the standard criterion for identification, *F. tularensis* is a fastidious organism making culture recovery a challenge, especially when analyzing animal carcasses. Tissues from dead animals are often overgrown with normal flora and other environmental contaminants. Past studies with carcasses have had limited success, and the culture recovery rates were approximately 30% (*19*). We achieved a culture recovery rate of 89.7% from *F. tularensis*–infected animals (both live and dead animals), demonstrating the sensitivity and usefulness of culture. In light of our findings, we suggest that culture on CHAB media containing antibiotics be attempted more routinely for diagnosis of *F. tularensis* infection in animal and field specimens such as water, mud, and grass or hay.

Additionally, when culture was used for detection of *F. tularensis* in animals that did not die of the disease, we found it more sensitive than either DFA (50%) or serologic testing (50%) and capable of detecting *F. tularensis* in all cases (100%). This high culture recovery rate is probably due to the freshness and relatively uncontaminated state of the specimens used for culture. The comparatively low detection levels of DFA and serologic testing were likely influenced by the fact that these animals were at varying stages of infection (acute phase and convalescent phase), making diagnosis by either DFA or serologic testing less than optimal. This suggests that for surveillance studies of *F. tularensis* infection in wild rodent populations, culture of fresh tissues is the preferred diagnostic method.

In contrast, in detecting *F. tularensis* in animals that died of the disease, DFA was more sensitive than culture (85.1%) and capable of detecting *F. tularensis* in all animals. For animals with fatal cases of tularemia in prairie dogs, the levels of *F. tularensis* were extremely high in both spleen and liver, simplifying identification by DFA. Culture recovery of *F. tularensis* was probably more difficult because of deterioration of the samples and loss of bacterial viability over time. Indeed, *F. tularensis* in the tissues of the seven presumptive-positive animals was noncultivatable and noninfectious as shown by passage of the tissues through Swiss-Webster mice.

Presumably, one or more *F. tularensis*–infected prairie dogs were among the thousands trapped and shipped to the Texas exotic animal facility. On arrival at the facility, the infected prairie dogs died. The bacterium was then transmitted throughout hundreds of prairie dogs at the facility most likely as the result of cannibalism. Several cases (Introduction and 7) of tularemia in prairie dogs have now been documented, suggesting that a proportion of wild prairie dogs harbor live *F. tularensis.* Environmental stresses, such as capture, transit, and crowding, may induce productive infection that manifests as severe disease and death. Given the seriousness of the resulting disease and the public health risk for pet-to-human transmission, long-term studies are needed to determine the length of time seropositive prairie dogs can harbor live *F. tularensis* and whether they are reservoirs of tularemia in nature.

## References

[1] Ellis J, Oyston CF, Green M, Titball RW. Tularemia. Clin Microbiol Rev. 2002;15:631–46. 10.1128/CMR.15.4.631-646.2002 12364373 PMC126859

[2] Centers for Disease Control and Prevention. Tularemia—United States, 1990–2000. MMWR Morb Mortal Wkly Rep. 2002;51:182–4.

[3] Dennis DT, Inglesby TV, Henderson DA, Barlett JG, Ascher MS, Eitzen E, Tularemia as a biological weapon. JAMA. 2001;285:2763–73. 10.1001/jama.285.21.2763 11386933

[4] Hopla CE, Hopla AK. Tularemia. In: Beran GW, Steele JH, editors. Handbook of zoonoses. 2nd ed. Boca Raton (FL): CRC Press, Inc.; 1994. p.113–26.

[5] Mörner T. The ecology of tularaemia. Rev Sci Tech. 1992;11:1123–30. 1305858

[6] Chu MC, Weyant R. *Francisella* and *Brucella*. In: Murray PR, Baron EJ, Jorgensen JH, Pfaller MA, Yolken RH, editors. Manual of clinical microbiology. 8th ed. Washington: American Society for Microbiology; 2003. p. 789–97.

[7] Sjöstedt A. Family XVII. *Francisellaceae*, Genus I. *Francisella*. In: Brenner DJ, editor. Bergey’s manual of systemic bacteriology. New York: Springer-Verlag. 2004; in press.

[8] La Regina M, Lonigro J, Wallace M. *Francisella tularens*is Infection in captive, wild-caught prairie dogs. Lab Anim Sci. 1986;36:178–80. 3702338

[9] Avashia SB, Petersen JM, Lindley CM, Schriefer ME, Gage KL, Cetron M, First reported prairie dog–to-human tularemia transmission, Texas, 2002. Emerg Infect Dis. 2004; 10. 15109417 10.3201/eid1003.030695 PMC3322778

[10] Johansson A, Berglund L, Eriksson U, Göransson I, Wollin R, Forsman M, Comparative analysis of PCR versus culture for diagnosis of ulceroglandular tularemia. J Clin Microbiol. 2000;38:22–6. 10618057 10.1128/jcm.38.1.22-26.2000 PMC86009

[11] Brown SL, McKinney FT, Klein GC, Jones WL. Evaluation of a safranin-O-stained antigen microagglutination test for *Francisella tularensis* antibodies. J Clin Microbiol. 1980;11:146–8. 6153660 10.1128/jcm.11.2.146-148.1980 PMC273341

[12] Centers for Disease Control and Prevention. Outbreak of tularemia among commercially distributed prairie dogs, 2002. MMWR Morb Mortal Wkly Rep 2002;51:688,699. 12233912

[13] Johansson A, Urich SK, Chu MC. Sj**ö**stedt A, Tärnvik A. In vitro susceptibility to quinolones of *Francisella tularensis* subspecies *tularensis.* Scand J Infect Dis. 2002;34:327–30. 10.1080/00365540110080773 12069013

[14] Jellison WL, Bell JF, Owen CR. Mouse disease studies. In: Beck JR, editor. The Oregon meadow mouse irruption of 1957–1958. Corvallis (OR): Federal Cooperative Extension Service/Oregon State College; 1958. p. 71–80.

[15] Vest ED, Marchette NJ. Transmission of *Pasteurella tularensis* among desert rodents through infective carcasses. Science. 1958;128:363–4. 10.1126/science.128.3320.363-a 13568797

[16] Hoogland JL. The black-tailed prairie dog: social life of a burrowing animal. Chicago: University of Chicago Press; 1995.

[17] Bell JF, Stewart SJ. Chronic shedding tularemia nephritis in rodents: possible relation to occurrence of *Francisella tularensis* in lotic waters. J Wildl Dis. 1975;11:421–30. 239255 10.7589/0090-3558-11.3.421

[18] Olsufjev NG, Shlygina KN, Ananova EV. Persistence of *Francisella tularensis* McCoy et Chapin tularemia agent in the organism of highly sensitive rodents after oral infection. J Hyg Epidemiol Microbiol Immunol. 1984;28:441–54. 6396330

[19] Mörner T, Sandström G, Mattsson R, Nilsson PO. Infections with *Francisella tularensis biovar palaearctica* in hares (*Lepus timidus, Lepus europaeus*) from Sweden. J Wildl Dis. 1988;24:10–4. 2900904 10.7589/0090-3558-24.3.422

